# Employment of an algorithm of care including chest physiotherapy results in reduced hospitalizations and stability of lung function in bronchiectasis

**DOI:** 10.1186/s12890-019-0844-4

**Published:** 2019-04-25

**Authors:** Jordan Powner, Andrew Nesmith, Denay P. Kirkpatrick, Jessica K. Nichols, Brent Bermingham, George M. Solomon

**Affiliations:** 1 University of Alabama at Birmingham Department of Medicine, Birmingham, AL USA; 2 Department of Medical Nursing, Birmingham, AL USA; 3 Gregory Fleming James Cystic Fibrosis Research Center, 1900 University Blvd THT 422, Birmingham, AL 35294 USA

**Keywords:** Bronchiectasis, Chest physiotherapy, High frequency chest wall oscillation, Mucociliary clearance, Treatment algorithm, Exacerbations, Lung function

## Abstract

**Background:**

There is a paucity of data on long term clinical effects of high frequency chest wall oscillation (HFCWO) in the Bronchiectasis population. Other therapies such as nebulized mucolytics and long term antibiotics have proven benefit on quality of life and exacerbation rate. In this study a treatment algorithm that included HFCWO as a component was initiated to see what the long term effects of the proposed algorithm were on lung function, antibiotic use, and exacerbation rates.

**Methods:**

This was an observational comparative retrospective cohort study from database of patients with Bronchiectasis. Patients with > 2 exacerbations and significant symptom burden were enrolled to receive a treatment algorithm. The algorithm included: nebulized bronchodilators, mucolytics (hypertonic saline (3–7%) or n-acetylcysteine) inhaled daily or twice daily, thrice weekly macrolide therapy when appropriate, and high frequency chest wall oscillation (HFCWO) therapy (daily to twice daily per issued protocol) Outcomes from the cohort were analyzed for the subsequent twelve months after initiation to observe longitudinal lung function and clinical outcomes. Chart review was then done to obtain data the year prior to the start of the algorithm in this same cohort of patients.

**Results:**

Sixty-five patients received the Smart Vest® HFCWO system and were enrolled into the algorithm for treatment during the study period. Of the sixty-five patients, forty-three were eligible due to adequate 1-year baseline and follow up data at the time of the study initiation. The mean FEV_1_ remained stable at 1-year post enrollment (1.85 ± 0.60 L pre vs 1.89 ± 0.60 L post, *p* = NS) and the number of exacerbations requiring hospitalization was reduced (1.3 ± 1.0 pre vs. 0.46 ± 0.81 hospitalizations, post initiation, *p* < 0.0001). Antibiotic use overall was also reduced (2.5 ± 0.86 courses/year pre vs 2.1 ± 0.92 courses per year post initiation, p < 0.0001).

**Conclusion:**

Standardized care for Bronchiectasis involving an algorithm for Mucociliary clearance that centers on initiation of HFCWO may help to reduce lung function decline, need for oral antibiotics, and reduced hospitalization rate.

## Background

Bronchiectasis is a disease state caused by a myriad of pathological processes. The phenotype of the disease remains largely unchanged with cough, sputum production, shortness of breath, and recurrent pulmonary infections. It is a disease that causes significant mortality and morbidity and is becoming more commonly diagnosed [1]. Awareness and diagnosis of the condition is growing, but treatment options remain limited [2]. Several treatment options that have been applied to Bronchiectasis caused by Cystic Fibrosis (CF) have been applied to Bronchiectasis with moderate success. Inhaled antibiotics such as tobramycin and colistin have been studied with improved outcomes on preventing exacerbations and treating bacterial infections [3, 4]. Long-term oral therapy with erythromycin was shown to decrease the number of exacerbations in a 12-month span, but also there remains concern about increased macrolide resistant in common pulmonary infections [5]. Another macrolide, azithromycin, in a similar study has also shown to reduce exacerbations in a 12-month time period with chronic therapy, but can also induce macrolide resistance especially in nontuberculosis mycobacteria infections [6]. A recent study concluded that prophylactic antibiotic therapy, either oral or inhaled, also decreased exacerbations in the frequent exacerbator phenotype; unfortunately it did not ameliorate exacerbations completely in this phenotype [7]. Therefore, a greater understanding of therapies is needed to improve clinical outcomes for Bronchiectasis.

Another main stay of therapy in CF Bronchiectasis is augmenting and improving mucociliary clearance. Mucociliary clearance therapy involves uses of nebulized medication such as albuterol, hypertonic saline, and rhDNase as well as chest physiotherapy via HFCWO, oscillatory positive expiratory pressure(PEP) device, or manual chest percussion [8, 9]. Expiratory therapies such as active cycle breathing with huff coughing and autogenic drainage have shown to increase mucous clearance in Bronchiectasis and other pulmonary diseases [10]. Inhaled medications rhDNase and hypertonic saline have had mixed success in Bronchiectasis [2]. Contrary to its effect in CF, rhDNase caused a significant decrease in FEV_1_ and had a higher rate of exacerbation [11]. Short term use (14 days) of rhDNase was also found to be ineffective on spirometry and quality of life [12]. Hypertonic saline in CF has been shown to decrease exacerbations and improve lung function [13, 14]. In Bronchiectasis, hypertonic saline has been shown to increase mucous clearance, quality of life, and lung function in the short-term [15]. Two different long term studies showed different effects of hypertonic saline on lung function and exacerbation rate in Bronchiectasis [16, 17]. One showed only improvement in quality of life and small airway dysfunction and another showed improvement in FEV_1_, FVC, and exacerbation rate. These studies provide a template for therapy for mucociliary clearance in Bronchiectasis while leaving the question of what more can be done?

Chest physiotherapy (CPT) with high frequency chest wall oscillation (HFCWO) has become an integral component of CF mucociliary clearance therapy in the US. In CF, vest physiotherapy has shown to provide therapeutic benefits in combination with the previous mentioned therapies [18]. HFCWO-based CPT has had multiple studies looking at sputum production and lung function with approximately half of the studies showing favorable results of vest therapy over other therapies for airway mucociliary clearance [9]. This makes HFCWO per vest therapy in CF a standard therapy, but there is minimal data to date for use in Bronchiectasis. One short term study in 2013 compared traditional chest physiotherapy to HFCWO using the Vest Airway Clearance System (Hill-Rom, Batesville, Indiana, USA) for 15 days and demonstrated a significant improvement in lung function with HFCWO [19]. Therapy with HFCWO compared to traditional techniques had greater improvements in FEV_1_ and FVC, sputum production, quality of life, and decreased inflammatory markers in the sputum over the 15 days of the study. This study however was not designed to look at long term outcomes in lung function or antibiotic use.

Given the very limited short- and long-term data on HFCWO for treatment in Bronchiectasis, we initiated a single center study to look at the efficacy of a treatment algorithm which included HFCWO. The primary endpoint was to determine the clinical effectiveness of a treatment algorithm centered on novel early initiation of HFCWO as an airway clearance treatment along with adjunctive nebulized bronchodilators, nebulized mucolytics, and macrolide therapy for patients with Bronchiectasis.

## Methods

The study protocol was approved by the IRB committee at the University of Alabama at Birmingham (UAB). Ethics approval and informed consent were waived by the IRB committee due to treatments being routine standard of care.

Patients were recruited for an observational comparative retrospective medical cohort study from the database of patients with Bronchiectasis in the UAB Adult Bronchiectasis Center. The diagnosis of Bronchiectasis was confirmed by high resolution computed tomography of the chest along with clinical symptoms of cough and sputum production. Patients were referred for advanced management of Bronchiectasis and were treated per the therapeutic algorithm if they reported greater than 2 exacerbations in the previous year and symptoms that warrant entry by the clinic staff. These symptoms included chronic cough, sputum production, or dyspnea. For study purposes an exacerbation was defined as patient reported or medical record evidence of: Increased cough/sputum production or change in chest imaging or change in lung function accompanied by treatment with antibiotics and/or corticosteroids. This was based off the consensus definition of a Bronchiectasis exacerbation as a deterioration in three or more of the.

following key symptoms for at least 48 h: cough; sputum volume and/or consistency; sputum purulence; breathlessness and/or exercise tolerance; fatigue and/or malaise; hemoptysis and a clinician determines that a change in bronchiectasis treatment is required [20]. In this study a severe exacerbation was classified as the necessity for a course of intravenous antibiotics or hospitalization for treatment.

The treatment algorithm (see Table 1**)**, consisted of a standardized treatment of nebulized bronchodilators (1 or more times daily), mucolytics (either hypertonic saline (3–7%) or n-acetylcysteine inhaled daily or twice daily), and thrice weekly macrolide therapy in appropriate patients with recurrent exacerbations. HFCWO therapy (daily to twice daily per issued protocol) was then initiated as the final treatment of the algorithm. The Minnesota protocol for smart vests was used in this study [21]. This standard protocol takes 30 min with pauses for patients to huff cough. After every pause the frequency and the pressure is increased to provide better clearance. Sixty-five patients qualified for treatment using this algorithm. Patients that did not qualify for the study were treated with usual care of nebulized therapies along with antibiotics as needed for exacerbations.

**Table 1:** Treatment Algorithm

> 2 Exacerbations per Year?	
If Yes	If No
1. Offer evaluation	Not included in Study
2. Start Nebulized Bronchodilator	
3. Start Nebulized Mucolytic	
4. Assess for Macrolide therapy	
5. Initiate High Flow Chest Wall Oscillation	

Patients were then followed in the outpatient clinical setting every 2–3 months for one year over the period of the study. The protocol involved clinic visits assessing patients clinically for exacerbations that required outpatient therapy with antibiotics and/or steroids, or hospitalization for treatment. Clinic visits also included spirometry to assess lung function as well as the need for above treatments. Pulmonary function (FVC, FEV_1,_ and FEF_25–75%_) was measured at each visit. The patients selected to receive therapy per the algorithm, including a HFCWO vest, were then assessed to see if they received and initiated treatment with the device and if there was sufficient longitudinal lung function and clinical outcome data to be included in longitudinal review. The patients who met criteria were then subjected to further chart review and complete records were obtained for each patient. Subsequent review included assessing the number of hospitalizations, antibiotic usage, and baseline lung function during the year prior to initiation of the algorithm.

Baseline lung function was defined as the mean of all values of the parameters over that previous year (minimum of 2 discrete values). All function values were obtained using ATS standards [22]. Percent predicted values were calculated for this mixed ethnicity population using the NHANESIII prediction rule.

Descriptive comparison statistics were used to compare the data obtained over the study period using the treatment algorithm to the data from the year prior to the initiation of the algorithm. Statistical analysis was done using Graphpad Prism 7.0 (GraphPad Software, La Jolla, CA). Given the non-parametric nature of the sample distribution, Wilcoxon and Mann-Whitney tests were used to compare group means pre- and post-treatment algorithm. Statistical significance was defined by a *p*-value less than 0.05.

## Results

In total, sixty-five patients were treated with the algorithm and received the Smart Vest HFCWO system during the study period and forty-three were eligible to be included in the study due to adequate 1-year baseline and follow up data at the time of the study initiation. The mean age was 60.7 years, and the patients were 78% female. Baseline FEV_1_ of the patients that were enrolled was 60%. Patient demographics along with the percentage of other bronchiectasis specific therapies the patients were on during the time of the study are detailed in Table 2. Of note, 19% of the patients had positive sputum for *Pseudomonas aeruginosa* during the study period.

**Table 2:** Patient Demographics

Mean age, yr. (S.D.)	60.7 (14.3)
Percentage Female (%)	78
Percentage Caucasian (%)	85%
Mean Baseline FEV_1_, L (S.D.)	1.85 (0.60)
Percentage of Patients Prescribed HTS	95%
Percentage of Patients Prescribed NAC	5%
Percentage of Patients Prescribed B.D.	100%
Percentage of Patients Prescribed Azithromycin	35%
Percentage of Patients Prescribed Inhalational Antibiotics	12%
Percent of Patients with at least one positive culture for P.a.	19%

Abbreviations: *yr*. years, *HTS* Hypertonic saline, *NAC* N-acetylcysteine, B.D. Bronchodilator, *P.a. Pseudomonas aeruginosa*

**Fig. 1:**
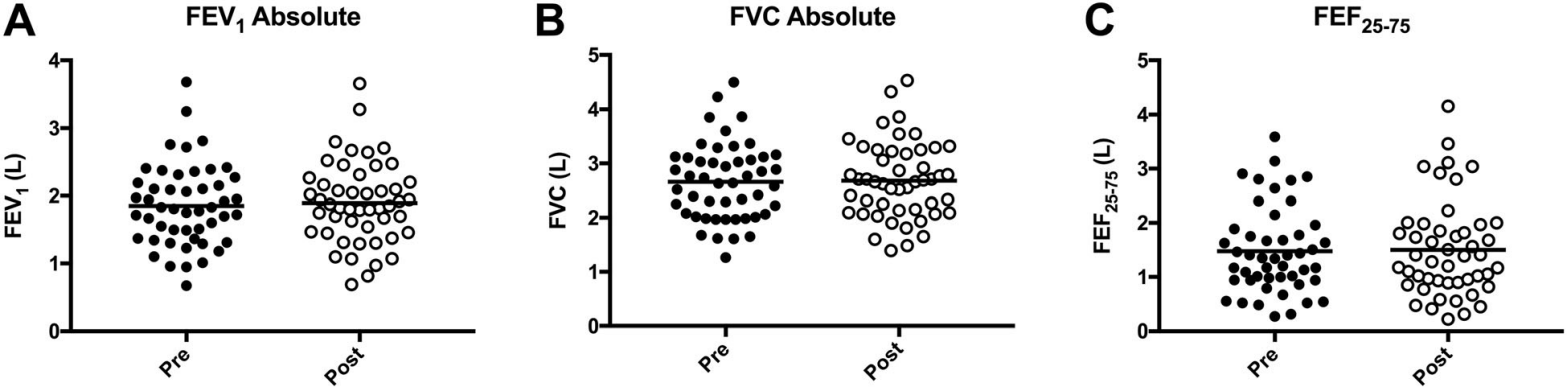
Mean lung function parameters pre and post-initiation of the algorithm including HFCWO in (**a**) FEV_1_, (**b**) FVC, and (**c**) mid-flows FEF_25–75_

The pre and post algorithm data on lung function can be seen in Fig. 1, which shows stability of lung function at one-year post algorithm-based treatment. The mean baseline FEV_1_ was 1.85 ± 0.60 L which at 1-year post treatment with the algorithm was 1.89 ± 0.60 L, the *p*-value comparing the two was not significant indicating it did not increase or decrease significantly. FVC and FEF_25–75%_ did not show significant change either (see Fig. 1). Out of forty-three patients, complete hospital records were obtained on thirty-nine patients providing an estimate on the number of severe exacerbations in the cohort pre and post-algorithm initiation. This provided enough data for an estimation of the effect the algorithm had on the amount of antibiotics prescribed and the number of severe exacerbations experienced in the cohort.

**Fig. 2:**
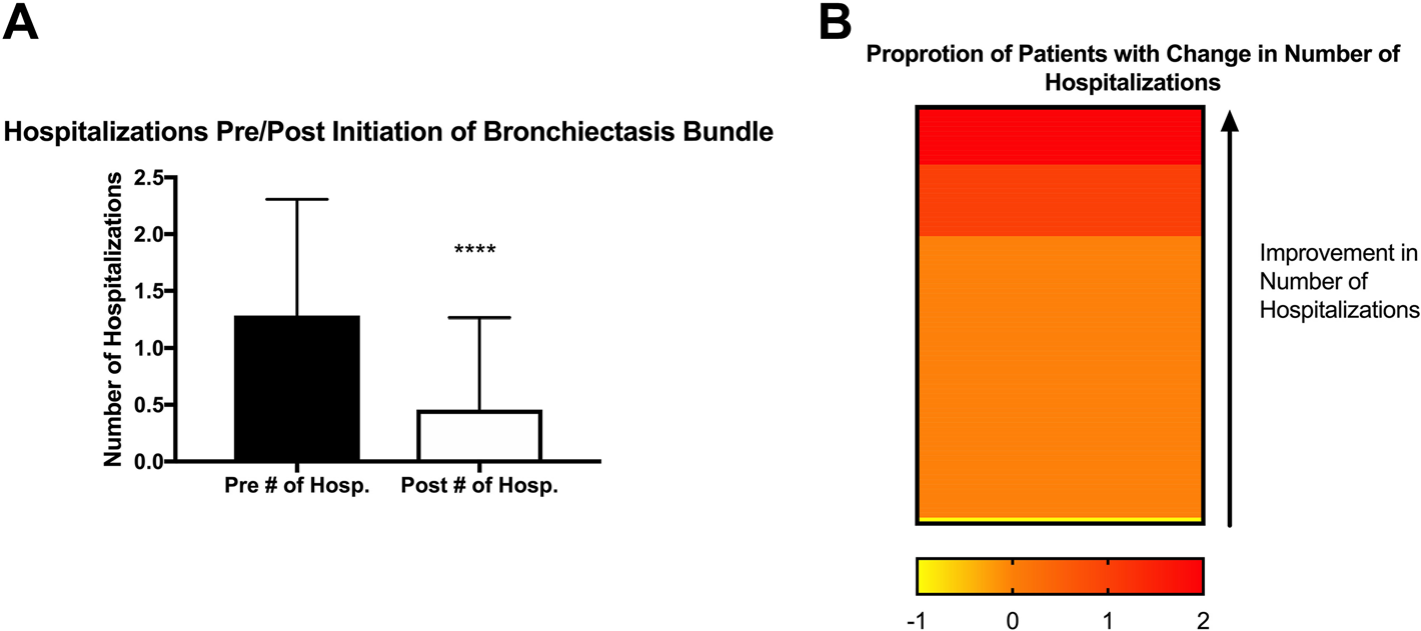
Effect of Algorithm therapy with HFCWO on exacerbations. (**a**) There was significant reduction in the number of exacerbations requiring hospitalization and (**b**) a minority of patients had increased hospitalizations (green line). ****p < 0.0001 by paired t-test (Wilcoxon)

The number of severe exacerbations decreased by a significant amount (1.3 ± 1.0 pre vs. 0.46 ± 0.81 hospitalizations, post initiation, *p* < 0.0001), see Fig. 2a. A small subset of patients did have an increased severe exacerbation rate, but was not significant. For analysis purposes a course of home intravenous antibiotics was considered a severe exacerbation and included in the hospitalization rate.

In this cohort, 33% of the patients had severe exacerbations pre-algorithm therapy with a significant reduction in the number post-algorithm therapy. Even though greater than 45% of the patients did not require hospitalization pre-algorithm or post-algorithm, as seen in Fig. 2b, this was still is a significant reduction in hospitalizations.

**Fig. 3:**
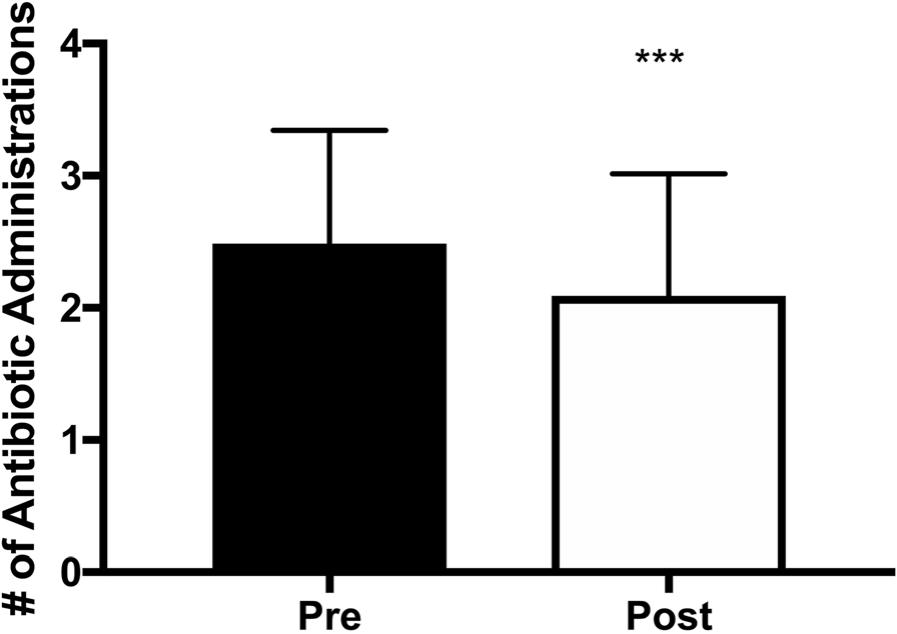
Antibiotic courses pre and post algorithm initiation. ****p < 0.0001 by paired t-test (Wilcoxon)

The number of courses of antibiotics decreased within the patient cohort after initiation of the algorithm (Fig. 3). The number of courses given decreased from 2.5 to 2.1 (*p* < 0.0005). While statistically significant, the clinical relevance of this modest reduction is unknown.

## Discussion

Therapy for Bronchiectasis is understudied due to low awareness and under-diagnosis. Current therapies only demonstrate modest reduction in symptoms, improvement in quality, reduction in exacerbations and hospitalizations, preservation of lung, and reduction in mortality [23]. Previous clinical reviews recommend airway clearance techniques, but only mention HFCWO showed benefit over PEP devices in one study [2, 24]. The British Thoracic Society does not include recommendations for HFCWO in use for Bronchiectasis [25]. The latest guidelines for Bronchiectasis were published by the European Respiratory Society and recommend chest physiotherapy that may include HFCWO in appropriate patients [26]. The endorsement for the use of HCWO is based on 1 short-term, small study which showed promise for the use of HFCWO in Bronchiectasis [19]. The Thoracic Society of Australia and New Zealand also do not mention or recommend the use of HFCWO in their guidelines, but do recommend airway clearance techniques [27]. Also, no studies involving HFCWO were included in the last comprehensive Cochrane review of treatments for bronchiectasis [28].

This is the first long-term study that involves a treatment algorithm for Bronchiectasis that involves standard therapies and standardized chest physiotherapy with HFCWO. These results demonstrate that a standardized treatment algorithm that includes HFCWO therapy can be a beneficial long-term treatment option for patients with Bronchiectasis to help improve symptom burden and quality of life. A significant reduction in hospitalizations was achieved in the study population being treated with the algorithm. The algorithm also decreased antibiotic use significantly and this may suggest an overall effective therapy in the prevention of all exacerbations and may show an even greater reduction in the frequent exacerbator phenotype. Unfortunately, the overall reduction in antibiotics may be modest in actuality and may not provide clinical significance.

Over the same year of therapy there was a stabilization of lung function. Neither the parameters of FEV_1_, FVC, FEF_25–75%_ showed any significant decline while on therapy. None of the other therapies included in Table 1. except hypertonic saline in limited studies improves lung function long term [16, 17]. This effect may be a consequence of the treatment algorithm as a whole or the long term initiation of HFCWO as a unique component.

Exacerbations can cause decreases in lung function and prolonged treatment with antibiotics that may require hospitalizations. This treatment algorithm may help prevent courses of antibiotics, worsening lung function, and hospitalizations. In frequent exacerbators, only macrolide therapy has previously been shown to reduce the overall number of exacerbations [5, 6]. The results obtained in this trial represent that the algorithm successfully decreased exacerbations and hospitalizations. Overall exacerbation declines may then translate into improved cost savings, quality of life, and lung function over time.

The potential reduction of hospitalizations is particularly impactful given the association of hospitalizations with increased mortality and morbidity in Bronchiectasis [29]. While it is a retrospective study the data shows promise for possible algorithmic care that includes HFCWO. The algorithm potentially decreases overall exacerbations, hospitalizations, and stabilizes lung function which could lead to cost-saving and improved quality of life in a debilitating condition. This was also shown in a patient group that was not sorted based on cause of Bronchiectasis but included patients with Bronchiectasis from any cause. Also, no patients were excluded due to known acute or chronic pulmonary infections or concurrent antibiotic use. The population in this study also included those considered to be the frequent exacerbator phenotype and did not exclude any patients based on any previous infections, reduced FEV_1_, radiographic severity, or overlapping COPD [7].

The weakness of any retrospective study includes biases that can be introduced during observation or analysis. There may have be effects on the outcomes due to the regular clinical visits by the patients and possible extra attention those enrolled may have received. In a single center there may be selection bias for patients and PI bias on the need to hospitalize patients for severe exacerbations. Exacerbations rates were also only measured by hospitalizations and antibiotic usage and mild exacerbations requiring increase in other therapies may have been missed. Compliance with the algorithm and its components could not be measured directly in between clinic appointments. Even though patients were provided chest vests and associated education, adherence to vest therapy and proper technique at home remains a question in this study. The sample size also does not allow analysis between distinct groups of Bronchiectasis or patients with different causes of Bronchiectasis or chronic infections. *Pseudomonas* infected patients were included in the cohort, meaning that this analysis also includes the frequent exacerbator phenotype. The study validates the need for a larger prospective study involving this therapeutic algorithm for Bronchiectasis.

## Conclusion

Taken together, this retrospective cohort demonstrates feasibility of data collection in a cohort of complex bronchiectasis patients receiving a treatment algorithm that includes HFCWO. The data also demonstrates the ability of this algorithm to help stabilize key lung function parameters and suggest reduction of the rate of decline of lung function. At the same time, the treatment algorithm was associated with significant reductions in the rate of exacerbations and hospitalizations which would also help slow the decline of lung function over time by preventing exacerbations. Despite smart technologies in our EMR, the retrospective nature of this study limits the ability to accurately report the overall effect on the frequencies of all-grade exacerbations. Again, this study was limited by its single center status, possible PI bias on the need for antibiotics, and retrospective nature. Outcomes could have also been influenced by the investigators knowledge of the patients in the database when evaluating them in clinic and also the regular clinic visits by the patients. Further studies would need to be multicenter and have a larger sample size to be able to control and analyze the individual components of the algorithm. Smart technology could also be used to determine the level of use and adherence of the vest by the participant.

## Abbreviations

CF: Cystic Fibrosis
HFCWO: High Frequency Chest Wall Oscillation
HTS: Hypertonic Saline
PEP: Positive Expiratory Pressure
